# Hormone receptor status of a first primary breast cancer predicts contralateral breast cancer risk in the WECARE study population

**DOI:** 10.1186/s13058-017-0874-x

**Published:** 2017-07-19

**Authors:** Anne S. Reiner, Charles F. Lynch, Julia S. Sisti, Esther M. John, Jennifer D. Brooks, Leslie Bernstein, Julia A. Knight, Li Hsu, Patrick Concannon, Lene Mellemkjær, Marc Tischkowitz, Robert W. Haile, Ronglai Shen, Kathleen E. Malone, Meghan Woods, Xiaolin Liang, Monica Morrow, Jonine L. Bernstein, on behalf of WECARE Study Collaborative Group

**Affiliations:** 1 Memorial Sloan Kettering Cancer Center, New York, NY USA; 2 University of Iowa, Iowa City, IA USA; 3 Cancer Prevention Institute of California, Fremont, CA USA; 4 Stanford Department of Medicine, Division of Oncology, and the Stanford Cancer Institute, Stanford, CA USA; 5 University of Toronto, Dalla Lana School of Public Health Sciences, Toronto, Canada; 6 Beckman Research Institute, City of Hope National Medical Center, Duarte, CA USA; 7 Lunenfeld-Tanenbaum Research Institute, Sinai Health System, Toronto, Canada; 8 Fred Hutchinson Cancer Research Center, Seattle, WA USA; 9 Genetics Institute and Department of Pathology, Immunology and Laboratory Medicine, University of Florida, Gainesville, FL USA; 10 Danish Cancer Society Research Center, Copenhagen, Denmark; 11 Department of Medical Genetics, University of Cambridge, Cambridge, UK; 12 MPH, 485 Lexington Avenue, 2nd Floor, New York, NY 10017 USA

**Keywords:** Contralateral breast cancer, Hormone receptor, Estrogen receptor, Progesterone receptor, Population-based

## Abstract

**Background:**

Previous population-based studies have described first primary breast cancer tumor characteristics and their association with contralateral breast cancer (CBC) risk. However, information on influential covariates such as treatment, family history of breast cancer, and *BRCA1/2* mutation carrier status was not available. In a large, population-based, case-control study, we evaluated whether tumor characteristics of the first primary breast cancer are associated with risk of developing second primary asynchronous CBC, overall and in subgroups of interest, including among *BRCA1/2* mutation non-carriers, women who are not treated with tamoxifen, and women without a breast cancer family history.

**Methods:**

The Women’s Environmental Cancer and Radiation Epidemiology Study is a population-based case-control study of 1521 CBC cases and 2212 individually-matched controls with unilateral breast cancer. Detailed information about breast cancer risk factors, treatment for and characteristics of first tumors, including estrogen receptor (ER) and progesterone receptor (PR) status, was obtained by telephone interview and medical record abstraction. Multivariable risk ratios (RRs) and 95% confidence intervals (CIs) were estimated in conditional logistic regression models, adjusting for demographics, treatment, and personal medical and family history. A subset of women was screened for *BRCA1*/2 mutations.

**Results:**

Lobular histology of the first tumor was associated with a 30% increase in CBC risk (95% CI 1.0–1.6). Compared to women with ER+/PR+ first tumors, those with ER-/PR- tumors had increased risk of CBC (RR = 1.4, 95% CI 1.1–1.7). Notably, women with ER-/PR- first tumors were more likely to develop CBC with the ER-/PR- phenotype (RR = 5.4, 95% CI 3.0–9.5), and risk remained elevated in multiple subgroups: *BRCA1*/2 mutation non-carriers, women younger than 45 years of age, women without a breast cancer family history, and women who were not treated with tamoxifen.

**Conclusions:**

Having a hormone receptor negative first primary breast cancer is associated with increased risk of CBC. Women with ER-/PR- primary tumors were more likely to develop ER-/PR- CBC, even after excluding *BRCA1/2* mutation carriers. Hormone receptor status, which is routinely evaluated in breast tumors, may be used clinically to determine treatment protocols and identify patients who may benefit from increased surveillance for CBC.

**Electronic supplementary material:**

The online version of this article (doi:10.1186/s13058-017-0874-x) contains supplementary material, which is available to authorized users.

## Background

Among women with a first breast cancer, risk of a second primary tumor in the contralateral breast is greater than risk of a first primary breast cancer in the general female population, [1–3] and contralateral breast cancer (CBC) represents the most frequent new malignancy diagnosed among breast cancer survivors [4]. The 25-year cumulative risk of CBC in the USA is approximately 7%, [4] with some evidence that CBC incidence has recently declined [5]. However, increases in breast cancer incidence, coupled with advances in treatment and improved survival, have led to a rise in the number of breast cancer survivors at risk of CBC. Identified risk factors for CBC include young age at first breast cancer diagnosis, [2, 6] breast cancer family history, [1, 7–10] mutations in *BRCA1* and *BRCA2* (*BRCA1/2),* [11, 12] young age at menarche, [13] nulliparity, [13] and obesity [14]. We and others have shown that both chemotherapy [15, 16] and tamoxifen therapy [16, 17] are associated with reduced CBC risk, although the benefit of tamoxifen on CBC risk was limited to estrogen receptor-positive (ER+) disease in the EBCTCG meta-analysis [17]. We further demonstrated that current users of tamoxifen with longer durations of use have the largest CBC reduction, which adds further support for the recent recommendations for primary breast cancer [18].

Additionally, some features of first breast tumors may predict CBC risk. Lobular histology of the first primary tumor has been associated with higher CBC incidence in several [1, 8, 9, 19, 20] although not all studies [6, 21, 22]. Evidence of associations with other histologic parameters remains equivocal [3, 23, 24]. Women with estrogen receptor-negative (ER-) first primary tumors, particularly those diagnosed at younger ages, may be at increased risk of CBC, and appear more likely to have second tumors that are ER- and high grade, [23, 25–29] potentially reflecting a higher number of *BRCA1* mutation carriers in this group [30, 31]. ER and progesterone receptor (PR) status, collectively referred to as hormone receptor (HR) status, is routinely evaluated to inform treatment decisions, and could potentially be used to identify women at increased CBC risk. Tumors that lack expression of HR (HR-), particularly those displaying the triple-negative phenotype, are associated with poorer clinical outcomes and presently there are fewer therapeutic options for these tumors than for HR-positive (HR+) and HER2-overexpressing tumors (HER2+) [32].

Several previous studies have examined characteristics of first breast tumors in relation to CBC risk, using cancer registry data, primarily the US National Cancer Institute’s Surveillance, Epidemiology, and End Results (SEER) Registry Program. Despite large sample sizes, these studies generally lacked detailed information on potentially important covariates including *BRCA1/2* mutation carrier status, breast cancer family history, and treatment for first breast cancer [25, 26, 28, 29]. Here, we evaluate first primary breast cancer characteristics and CBC risk in a population-based case-control study of CBC cases and unilateral breast cancer (UBC) controls.

## Methods

### Study population

The Women’s Environmental Cancer and Radiation Epidemiology (WECARE) Study is a multicenter, population-based case-control study in which UBC controls were individually matched to (cases) with asynchronous CBC. The study design of the first phase (WECARE I Study) has been described in detail elsewhere; [33] the second phase (WECARE II Study) employed a nearly identical approach [16]. Briefly, participants were identified through eight population-based cancer registries: six in the USA, one in Canada, and one in Denmark (Table 1). The study protocol was approved by the institutional review boards at each site and the Ethics Committee System in Denmark.

**Table 1:** Characteristics of cases and controls from the WECARE Study population

Variable	CBC cases *N* = 1521	UBC controls *N* = 2212
Age at first diagnosis (years), median (range)	46 (24-54)	46 (23-54)
Age at reference date (years), median (range)	53 (27-73)	52 (27-71)
Length of at-risk period (years)^a^, median (range)	6.3 (1.0-19.8)	5.5 (1.0-19.8)
Study area, N (%)		
Iowa^b^ California^c^ Seattle^d^ Denmark^e^ Canada^f^	201 (13) 658 (43) 224 (15) 279 (18) 159 (10)	314 (14) 967 (44) 317 (14) 457 (21) 157 (7)
Year of first breast cancer diagnosis, N (%)		
1985–1988 1989–1992 1993–1996 1997–2008	238 (16) 415 (27) 427 (28) 441 (29)	467 (21) 647 (29) 632 (29) 466 (21)
Age at menarche (years), N (%)		
Never had menses <13 ≥13 Unknown	3 (0) 724 (48) 791 (52) 3 (0)	6 (0) 965 (44) 1239 (56) 2 (0)
Number of full-term pregnancies, N (%)		
None 1 2 3 ≥4 Unknown	322 (21) 271 (18) 559 (37) 256 (17) 108 (7) 5 (0)	412 (19) 341 (15) 842 (38) 387 (17) 225 (10) 5 (0)
Menopausal status^g^, N (%)		
Premenopausal Postmenopausal Unknown	1124 (74) 389 (26) 8 (1)	1676 (76) 522 (24) 14 (1)
First-degree family history of breast cancer, N (%)		
No Yes Adopted/unknown	1004 (66) 497 (33) 20 (1)	1706 (77) 466 (21) 40 (2)
Stage of first diagnosis, N (%)		
Local Regional Unknown	1061 (70) 448 (29) 12 (1)	1442 (65) 759 (34) 11 (1)
Chemotherapy, N (%)		
No Yes	699 (46) 822 (54)	923 (42) 1289 (58)
Radiation treatment, N (%)		
No Yes Unknown	641 (42) 880 (58) 0 (0)	522 (24) 1689 (76) 1 (0)
*BRCA1/2* deleterious mutations, N (%)		
No	596 (39)	1322 (60)
Yes	109 (7)	76 (3)
Not tested^h^	816 (54)	814 (37)
Hormone treatment, N (%)		
No Yes Unknown	964 (63) 557 (37) 0 (0)	1270 (57) 940 (42) 2 (0)

*Abbreviations*: *WECARE* Women’s Environmental Cancer and Radiation Epidemiology Study, *CBC* contralateral breast cancer, *UBC* unilateral breast cancer. ^a^Beginning at least one year after first diagnosis and extending to the date of CBC diagnosis of cases. ^b^The State Health Registry of Iowa. ^c^Four study centers: (1) Los Angeles County Cancer Surveillance Program, (2) The Cancer Surveillance Program of Orange County/San Diego-Imperial Organization for Cancer Control, (3) Greater Bay Area Cancer Registry (San Francisco Bay Area Region and Santa Clara Region), and (4) Sacramento and Sierra Center Registry (Sacramento Region). ^d^Cancer Surveillance System of the Fred Hutchinson Cancer Research Center. ^e^The Danish Breast Cancer Cooperative Group Database supplemented by the Danish Cancer Registry. ^f^The Ontario Cancer Registry. ^g^Women were classified as premenopausal if they reported having menstrual periods or being pregnant within 2 years of initial diagnosis. ^h^Only WECARE I Study participants were screened for *BRCA1/2* deleterious mutations

Cases were participants who were: (1) diagnosed between 1985 and 2008 with a first invasive breast cancer that had not spread beyond regional lymph nodes at diagnosis and a second contralateral primary breast cancer at least 1 year after the first diagnosis; (2) younger than 55 years at first diagnosis; (3) without previous or intervening cancer diagnosis except non-melanoma skin cancer or cervical carcinoma in situ; (4) alive at contact; (5) willing to provide informed consent and a biospecimen; and (6) residents of the same cancer registry reporting region for both diagnoses. Controls with an intact contralateral breast were identified using the same eligibility criteria, and individually matched to cases (1:2 in the WECARE I Study; 1:1 in the WECARE II Study) on the following criteria: diagnosis age (5-year strata), diagnosis year (4-year strata), cancer registry region, and race/ethnicity. To improve statistical efficiency, WECARE I Study cases and controls were additionally counter-matched on cancer-registry-reported treatment with radiation such that two members of the case-control triad had received radiation therapy for their index breast cancer.

We identified a total of 2354 CBC (cases) and 3599 UBC controls eligible for the study. Of those, 1521 patients and 2212 controls provided written informed consent, completed the interview, and provided a biospecimen.

### Data collection

Study participants were interviewed by telephone using a structured questionnaire to evaluate known or suspected breast cancer risk factors, including demographics, medical history, reproductive history, breast cancer family history, hormone use, smoking and alcohol intake. Detailed data on treatment, tumor characteristics including HR status were abstracted from pathology and surgical reports, radiation oncology clinic notes, and systemic adjuvant treatment reports. Information on tumor characteristics was also obtained from SEER registry records for US participants and from the Danish Breast Cancer Cooperative Group records for Danish participants. HER2 status was available only for WECARE II Study participants. ER, PR and HER2 status were each reported as “positive” (+), “negative” (-) or “unknown”. Self-reported treatment data were used for participants with missing information in their medical records (chemotherapy, 4%; hormonal therapy, 5%).

Participants in the WECARE I Study were screened for *BRCA1/2* mutations using denaturing high-performance liquid chromatography [34]. Carriers of *BRCA1/2* mutations were defined based on the presence of variants known or predicted to truncate the protein including frameshifts and premature termination codons, mutations occurring within 2 bp of an intron/exon boundary, and missense substitutions known to have deleterious functional effects.

### Statistical analysis

Data from the two study phases were combined for the analyses presented here. Multivariable conditional logistic regression models were fit to estimate adjusted risk ratios (RRs) and corresponding 95% confidence intervals (CIs). Models included the following known or suspected CBC risk factors: age at first breast cancer diagnosis, breast cancer family history, age at menarche, parity, menopausal status, lobular histology of first breast cancer, and treatment for first breast cancer (hormonal, radiation therapy, chemotherapy). To account for the counter-matched design of the WECARE I Study, models included a log-weight covariate. WECARE II Study participants (who were not counter-matched) were assigned an offset term of 1 [33]. During the study period, clinical guidelines for tamoxifen evolved, which is reflected in the reporting of this treatment in our study. In 1988, tamoxifen was recommended for women with lymph-node-negative breast cancer [35]. A decade later, guidelines for women with ER+ breast cancer recommended adjuvant tamoxifen for 5 years [36, 37]. We chose cut points of <5, 5–9, and ≥10 years for analysis by time since the first breast cancer diagnosis and further adjusted these estimates for tumor stage in view of changing tamoxifen guidelines during the study period. Previous work demonstrated that nearly all tumors classified as ER-/PR+ in medical records are re-classified by either immunohistochemical analysis or gene expression analysis; [38] therefore, RR estimates for first primary tumors identified as ER-/PR+ are not reported in HR status analyses. We evaluated associations of HR status of the first breast tumor with risk of ER+/PR+, ER+/PR- and ER-/PR- CBC. We examined CBC risk among 705 cases and 1398 controls who were tested for *BRCA1/2* deleterious mutations, and repeated the analysis excluding *BRCA1/2* mutation carriers. The association of HR status of the first breast tumor with CBC risk and subtype-specific CBC was additionally adjusted for *BRCA1* deleterious mutation status in a post-hoc analysis. Likelihood ratio tests were utilized to assess heterogeneity for potential effect modifiers, including differences in all tumor characteristics and tamoxifen between the WECARE I and WECARE II Studies (all *P*_heterogeneity_ by study phase ≥0.17). Analyses were conducted using SAS v9.4 (SAS Institute, Cary, NC, USA).

## Results

Our analysis includes 1521 CBC cases and 2212 UBC controls. There were 2107 participants in the WECARE I Study and 1626 participants in the WECARE II Study. Median age at first diagnosis was 46 years and median time to CBC diagnosis among cases was 6.3 years (Table 1). Overall, approximately 75% of participants were premenopausal at first diagnosis.

Lobular histology of the first breast cancer was associated with elevated CBC risk (RR = 1.3, 95% CI 1.0–1.6) (Table 2). This association was limited to non-users of tamoxifen (users RR = 1.0, 95% CI 0.7–1.4; non-users RR = 1.6, 95% CI 1.1–2.1, *P*_heterogeneity_ = 0.045). Other features of the first tumor, including grade, stage, nodal involvement, and tumor size, were not associated with CBC risk.

**Table 2:** Association between tumor characteristics of first primary breast cancer and contralateral breast cancer risk

First primary breast cancer	Cases, *N* (%)	Controls, *N* (%)	RR^*a*^ (95% CI)
Histology			
Non-lobular	1338 (88)	1986 (90)	1.0 (ref.)
Lobular	179 (12)	223 (10)	1.3 (1.0, 1.6)
Unknown	4 (0)	3 (0)	
Grade			
Well	248 (16)	338 (15)	1.0 (ref.)
Moderate	417 (27)	688 (31)	0.8 (0.7, 1.1)
Poor/undifferentiated	503 (33)	640 (29)	1.1 (0.8, 1.4)
Unknown	353 (23)	546 (25)	
Stage			
Localized	1061 (70)	1442 (65)	1.0 (ref.)
Regional	448 (29)	759 (34)	1.0 (0.9, 1.2)
Unknown	12 (1)	11 (1)	
Lymph node status			
Negative	1045 (69)	1426 (64)	1.0 (ref.)
Positive	438 (29)	751 (34)	1.0 (0.8, 1.2)
No nodes sampled	22 (2)	26 (1)	1.3 (0.7, 2.4)
Unknown	16 (1)	9 (0)	
Tumor size			
≤10 mm	335 (22)	510 (23)	1.0 (ref.)
>10 to ≤20 mm	595 (39)	913 (41)	1.1 (0.9, 1.3)
>20 mm	477 (31)	662 (30)	1.1 (0.9, 1.4)
Inflammatory	6 (0)	2 (0)	
Unknown	108 (7)	125 (6)	
ER status			
Positive	797 (52)	1254 (57)	1.0 (ref.)
Negative	467 (31)	561 (25)	1.3 (1.1, 1.6)
Other/unknown^b^	257 (17)	397 (18)	
PR status			
Positive	687 (45)	1083 (49)	1.0 (ref.)
Negative	442 (29)	549 (25)	1.2 (1.0, 1.5)
Other/unknown^b^	392 (26)	580 (26)	
Joint ER/PR status			
ER+/PR+	621 (41)	958 (43)	1.0 (ref.)
ER+/PR-	85 (6)	142 (6)	1.0 (0.7, 1.4)
ER-/PR-	352 (23)	387 (18)	1.4 (1.1, 1.7)
Other/unknown^b,c^	463 (30)	725 (33)	
HER2 status^d^			
Negative	207 (25)	222 (27)	1.0 (ref.)
Positive	53 (7)	63 (8)	0.8 (0.5, 1.3)
Unknown^b^	553 (68)	528 (65)	
Triple-negative^d^			
No	514 (63)	579 (71)	1.0 (ref.)
Yes	59 (7)	40 (5)	1.3 (0.8, 2.2)
Other/unknown^b^	240 (30)	194 (24)	

*Abbreviations*: *RR* risk ratio, *CI* confidence interval, *N* number, *ER* estrogen receptor, *PR* progesterone receptor, *HER2* human epidermal growth factor receptor 2. ^a^Adjusted for age at first breast cancer diagnosis, first-degree family history of breast cancer, histologic assessment, menopausal status, age at menarche, parity, radiation, chemotherapy and hormone therapy at first breast cancer diagnosis. Model examining histology was not adjusted for histologic assessment. ^b^“Other/unknown” category consists of women for whom no laboratory test was given, the test was given and the results were unknown or the test was given and the results were borderline; estimates not reported. The start date for ER/PR reporting in SEER was 1 January 1990 [47]. ^c^Includes 59 cases and 108 controls classified as ER-/PR+. ^d^Among participants in the Women’s Environmental Cancer and Radiation Epidemiology II Study (WECARE II) only; HER2 status not queried for WECARE I participants. Surveillance, Epidemiology, and End Results (SEER) Registry only began collecting HER2 status in 2010 [46]

Women whose first primary tumor was HR- had a higher risk of CBC than women with HR+ disease (Table 2). ER- first tumor status was associated with a 30% (95% CI 1.1–1.6) increase in CBC risk compared to ER+ first tumors; similarly elevated risks were observed when comparing PR- to PR+ first breast cancers (RR = 1.2, 95% CI 1.0–1.5). When ER and PR were evaluated jointly, ER-/PR- first tumor status was associated with a higher CBC risk than ER+/PR+ status of first tumors (RR = 1.4, 95% CI 1.1–1.7). Overall results for HR status were not appreciably different in the subset of women who had not received tamoxifen for their first diagnosis (1054 cases/1425 controls; see Additional file 1). Among WECARE II Study participants who were tested for HER2 status, neither HER2 positivity (RR = 0.8, 95% CI 0.5–1.3) nor the triple-negative phenotype (ER-/PR-/HER2-) was statistically significantly associated with CBC risk (RR = 1.3, 95% CI 0.8–2.2).

Although there was no statistically significant heterogeneity, for HR- tumors, the increased CBC risk was greatest in the first 10 years following the first breast cancer diagnosis, with no association seen among women who were diagnosed with CBC more than 10 years after their first diagnosis (<5 years ER-/PR- vs. ER+/PR+ RR = 1.5, 95% CI 1.1–2.1; ≥10 years ER-/PR- vs. ER+/PR+ RR = 1.1, 95% CI 0.7–1.6) (Table 3). Similar patterns according to time since first diagnosis were observed among women who had not used tamoxifen for their first breast cancer. The associations between HR status of first tumor and CBC risk were not modified by either first-degree breast cancer family history (any family history ER-/PR- vs. ER+/PR+ RR = 1.5, 95% CI 1.1–2.3; no family history RR = 1.3, 95% CI 1.0–1.7, *P*_heterogeneity_ = 0.43) or diagnosis age (<45 years ER-/PR- vs. ER+/PR+ RR = 1.4, 95% CI 1.0–1.8; ≥45 years RR = 1.3, 95% CI 1.0–1.7, *P*_heterogeneity_ = 0.79).

**Table 3:** Association between HR status of first breast cancer and CBC risk, by time since diagnosis

	Time <5 years to CBC		Time 5 to <10 years to CBC		Time ≥10 years to CBC		
	*N* (cases/controls)	RR^a^ (95% CI)	*N* (cases/controls)	RR^a^ (95% CI)	*N* (cases/controls)	RR^a^ (95% CI)	*P*-heterogeneity
All women, tumor status at first diagnosis							
ER status							
Positive	306/585	1.0 (ref.)	293/440	1.0 (ref.)	198/229	1.0 (ref.)	
Negative	186/241	1.4 (1.1, 1.9)	186/209	1.3 (1.0, 1.8)	95/111	1.0 (0.7, 1.5)	0.37
Other/unknown^b^	94/160		95/155		68/82		
PR status							
Positive	262/477	1.0 (ref.)	249/395	1.0 (ref.)	176/211	1.0 (ref.)	
Negative	183/238	1.3 (1.0, 1.8)	169/203	1.3 (1.0, 1.8)	90/108	0.9 (0.6, 1.4)	0.26
Other/unknown^b^	141/271		156/206		95/103		
Joint ER/PR status							
ER+/PR+	239/423	1.0 (ref.)	219/351	1.0 (ref.)	163/184	1.0 (ref.)	
ER+/PR-	38/68	1.0 (0.6, 1.6)	31/48	1.2 (0.7, 2.2)	16/26	0.6 (0.3, 1.3)	
ER-/PR-	145/166	1.5 (1.1, 2.1)	136/145	1.5 (1.0, 2.0)	71/76	1.1 (0.7, 1.6)	0.45
Other/unknown^b,c^	164/329		188/260		111/136		
Women who did not receive tamoxifen treatment for first diagnosis, tumor status at first diagnosis							
ER status							
Positive	169/290	1.0 (ref.)	141/196	1.0 (ref.)	97/108	1.0 (ref.)	
Negative	169/212	1.4 (0.9, 2.1)	166/173	1.4 (0.9, 2.0)	87/92	1.1 (0.7, 1.9)	0.77
Other/unknown^b^	85/142		80/135		60/77		
PR status							
Positive	135/222	1.0 (ref.)	117/165	1.0 (ref.)	84/102	1.0 (ref.)	
Negative	159/187	1.4 (0.9, 2.1)	145/160	1.3 (0.8, 2.0)	82/82	1.3 (0.7, 2.2)	0.97
Other/unknown^b^	129/235		125/179		78/93		
Joint ER/PR status							
ER+/PR+	121/185	1.0 (ref.)	97/136	1.0 (ref.)	75/83	1.0 (ref.)	
ER+/PR-	24/31	1.3 (0.6, 2.7)	16/26	0.7 (0.3, 2.0)	12/13	1.6 (0.4, 6.2)	
ER-/PR-	135/152	1.4 (0.9, 2.3)	127/125	1.5 (0.9, 2.4)	67/64	1.3 (0.7, 2.4)	0.84
Other/unknown^b,c^	143/276		147/217		90/117		

*Abbreviations*: *HR* hormone receptor, *CBC* contralateral breast cancer, *RR* risk ratio, *CI* confidence interval, *N* number, *ER* estrogen receptor, *PR* progesterone receptor

^a^Adjusted for age at first breast cancer diagnosis, first-degree family history of breast cancer, histologic assessment, stage, menopausal status, age at menarche, parity, radiation, chemotherapy and hormone therapy at first breast cancer diagnosis. ^b^“Other/unknown” category consists of women for whom no laboratory test was given, the test was given and the results were unknown or the test was given and the results were borderline; estimates not reported. ^c^Includes tumors classified as ER-/PR+

Women diagnosed with an ER-/PR- first breast cancer were less likely than women with an ER+/PR+ tumor to develop ER+/PR+ CBC (RR = 0.7, 95% CI 0.5–1.0) (Table 4). In contrast, the risk of developing an ER-/PR- CBC was fivefold greater among women with ER-/PR- first breast cancer than among those with ER+/PR+ first cancer (RR = 5.4, 95% CI 3.0–9.5). There was no effect modification by age, family history, or tamoxifen therapy; elevated RRs of ER-/PR- CBC following ER-/PR- first breast cancer were also observed for women <45 years of age at first diagnosis (RR = 5.9, 95% CI 2.9–12.2), those without family history of breast cancer (RR = 5.2, 95% CI 2.8–9.7) and those who were not treated with tamoxifen (RR = 6.5, 95% CI 3.2–12.9).

**Table 4:** Association between hormone receptor status of first breast cancer and subtype-specific contralateral breast cancer

	ER+/PR+ CBC		ER+/PR- CBC		ER-/PR- CBC	
Tumor status at first diagnosis	*N* (cases/controls)	RR^a^ (95% CI)	*N* (cases/controls)	RR^a^ (95% CI)	*N* (cases/controls)	RR^a^ (95% CI)
ER status						
Positive	365/421	1.0 (ref.)	123/147	1.0 (ref.)	89/241	1.0 (ref.)
Negative	95/182	0.6 (0.4, 0.9)	55/67	0.9 (0.5, 1.6)	187/112	3.9 (2.4, 6.5)
Other/unknown^b^	74/80		16/42		31/55	
PR status						
Positive	321/386	1.0 (ref.)	109/130	1.0 (ref.)	81/214	1.0 (ref.)
Negative	106/184	0.8 (0.6, 1.1)	52/63	0.9 (0.5, 1.5)	183/115	3.8 (2.3, 6.3)
Other/unknown^b^	107/113		33/63		43/79	
Joint ER/PR status						
ER+/PR+	307/343	1.0 (ref.)	97/112	1.0 (ref.)	64/193	1.0 (ref.)
ER+/PR-	30/50	0.7 (0.4, 1.2)	14/15	1.2 (0.5, 2.7)	20/28	2.6 (1.1, 5.7)
ER-/PR-	74/130	0.7 (0.5, 1.0)	38/48	0.8 (0.4, 1.5)	162/83	5.4 (3.0, 9.5)
Other/unknown^b,c^	123/160		45/81		61/104	

*Abbreviations*: *CBC* contralateral breast cancer, *RR* risk ratio, *CI* confidence interval, *N* number, *ER* estrogen receptor, *PR* progesterone receptor

^a^Adjusted for age at first breast cancer diagnosis, first-degree family history of breast cancer, histologic assessment, menopausal status, age at menarche, parity, radiation, chemotherapy and hormone therapy at first breast cancer diagnosis. ^b^“Other/unknown” category consists of women for whom no laboratory test was given, the test was given and the results are unknown or the test was given and the results were borderline; estimates not reported. ^c^Includes tumors classified as ER-/PR+

Among the women screened for *BRCA1/2* mutations, 185 (109 cases, 76 controls) were mutation carriers. Excluding these women attenuated the increased RRs for CBC observed among all women when comparing HR+ to HR- first breast cancers, but the increased risk of ER-/PR- CBC following a diagnosis of an ER-/PR- first tumor, compared to an ER+/PR+ first tumor, persisted (all tested women RR = 7.6, 95% CI 3.0–19.5; non-carriers RR = 7.7, 95% CI 2.6–23.3) (Table 5). The findings were similar when *BRCA1* deleterious mutation status was included in the multivariable model (all tested women RR = 7.6, 95% CI 3.0–19.5; all tested women adjusting for BRCA1 deleterious mutation status RR = 8.9, 95% CI 3.1–25.9). Excluding only *BRCA1* carriers yielded an effect of similar magnitude for the risk of ER-/PR- CBC following an ER-/PR- first primary (results not shown).

**Table 5:** Association between first breast cancer HR status and subtype-specific CBC, among known *BRCA1/2* mutation non-carriers

	Overall CBC			ER+/PR+ CBC			ER+/PR- CBC			ER-/PR- CBC		
	*N* (cases/controls)	RR^a^ (95% CI)	RR^b^ (95% CI)	*N* (cases/controls)	RR^a^ (95% CI)	RR^b^ (95% CI)	*N* (cases/controls)	RR^a^ (95% CI)	RR^b^ (95% CI)	*N* (cases/controls)	RR^a^ (95% CI)	RR^b^ (95% CI)
All women tested for *BRCA1/2* mutations^c^, tumor status at first diagnosis												
ER status												
Positive	336/745	1.0 (ref.)	1.0 (ref.)	100/179	1.0 (ref.)	1.0 (ref.)	41/66	1.0 (ref.)	1.0 (ref.)	26/114	1.0 (ref.)	1.0 (ref.)
Negative	193/338	1.4 (1.1, 1.9)	1.2 (0.9, 1.6)	22/82	0.6 (0.3, 1.2)	0.7 (0.3, 1.3)	15/31	0.8 (0.3, 2.0)	0.8 (0.3, 2.0)	56/56	4.6 (2.1, 10.2)	3.8 (1.6, 8.9)
Other/unknown^d^	176/315			33/44			9/30			19/32		
PR status												
Positive	287/615	1.0 (ref.)	1.0 (ref.)	88/160	1.0 (ref.)	1.0 (ref.)	35/61	1.0 (ref.)	1.0 (ref.)	23/95	1.0 (ref.)	1.0 (ref.)
Negative	172/318	1.3 (1.0, 1.7)	1.1 (0.8, 1.5)	26/86	0.8 (0.4, 1.5)	0.8 (0.5, 1.5)	16/20	2.2 (0.7, 6.8)	2.2 (0.7, 6.8)	56/55	5.3 (2.3, 12.2)	6.8 (2.6, 17.6)
Other/unknown^d^	246/465			41/59			14/46			22/52		
Joint ER/PR status												
ER+/PR+	250/536	1.0 (ref.)	1.0 (ref.)	84/143	1.0 (ref.)	1.0 (ref.)	29/46	1.0 (ref.)	1.0 (ref.)	16/86	1.0 (ref.)	1.0 (ref.)
ER+/PR-	38/85	1.1 (0.7, 1.7)	1.1 (0.7, 1.7)	9/22	0.9 (0.3, 2.3)	0.8 (0.3, 2.2)	7/4	4.9 (0.9, 26.5)	5.0 (0.9, 27.3)	8/14	4.6 (1.3, 16.4)	5.3 (1.3, 21.5)
ER-/PR-	132/221	1.5 (1.1, 2.1)	1.2 (0.9, 1.7)	17/61	0.7 (0.3, 1.5)	0.8 (0.4, 1.6)	9/16	1.2 (0.3, 4.7)	1.2 (0.3, 4.6)	48/41	7.6 (3.0, 19.5)	8.9 (3.1, 25.9)
Other/unknown^d.e^	285/556			45/79			20/61			29/61		
*BRCA1/2* non-carriers only, tumor status at first diagnosis												
ER status												
Positive	307/716	1.0 (ref.)		89/178	1.0 (ref.)		39/64	1.0 (ref.)		23/106	1.0 (ref.)	
Negative	137/304	1.2(0.9, 1.7)		19/70	0.7 (0.3, 1.5)		12/29	0.4 (0.1, 1.4)		33/52	3.6 (1.4, 8.9)	
Other/unknown^d^	152/302			30/42			9/29			14/32		
PR status												
Positive	261/593	1.0 (ref.)		81/159	1.0 (ref.)		32/59	1.0 (ref.)		18/88	1.0 (ref.)	
Negative	122/286	1.0 (0.8, 1.4)		21/75	0.8 (0.4, 1.7)		14/19	1.6 (0.5, 5.3)		36/52	6.7 (2.4, 18.9)	
Other/unknown^d^	213/443			36/56			14/44			16/50		
Joint ER/PR status												
ER+/PR+	233/520	1.0 (ref.)		77/143	1.0 (ref.)		27/45	1.0 (ref.)		15/80	1.0 (ref.)	
ER+/PR-	31/78	0.9 (0.5, 1.4)		7/22	0.7 (0.2, 2.0)		7/4	4.4 (0.8, 23.6)		6/13	3.8 (0.8, 17.2)	
ER-/PR-	90/197	1.2 (0.8, 1.8)		14/50	0.9 (0.4, 2.0)		7/15	0.6 (0.1, 2.8)		30/39	7.7 (2.6, 23.3)	
Other/unknown^d,e^	242/527			40/75			19/58			19/58		

*Abbreviations*: *HR*, hormone receptor, *CBC* contralateral breast cancer, *N* number, *RR* risk ratio, *CI* confidence interval, *ER* estrogen receptor, *PR* progesterone receptor. ^a^Adjusted for age at first breast cancer diagnosis, first-degree family history of breast cancer, histologic assessment, menopausal status, age at menarche, parity, radiation, chemotherapy and hormone therapy at first breast cancer diagnosis. ^b^Adjusted for all covariates listed in footnote a and in addition *BRCA1* deleterious mutation status. ^***c***^ *BRCA1/*2 mutation status only assessed among women sampled in the first phase of the Women’s Environmental Cancer and Radiation Epidemiology Study (WECARE) I. ^d^“Other/unknown” category consists of women for whom no laboratory test was given, the test was given and the results were unknown or the test was given and the results were borderline; estimates not reported. ^e^Includes tumors classified as ER-/PR+

## Discussion

In the WECARE Study, an HR- first breast cancer was associated with greater CBC risk than an HR+ first breast cancer. In particular, having a first tumor that lacked both ER and PR expression was associated with a more than fivefold greater risk of developing a CBC with the same HR-defined phenotype than first tumors that expressed both markers. Similar patterns of association between HR- first primary tumors and overall and subtype-specific CBC risk were observed when women treated with tamoxifen were excluded, suggesting that these associations were not due to lower CBC risk following tamoxifen treatment for ER+ first tumors. A lobular versus non-lobular histologic assessment at first diagnosis conferred a 30% increased CBC risk. Other features of first breast cancers were not associated with CBC risk.

Our analysis benefits from the collection of detailed data on treatment history and potential confounders, including reproductive and family history. Many previous analyses have relied on data from cancer registries, including SEER, which records only limited covariate data and intended first course treatment. Similar to our results, the preponderance of studies indicate that HR- first breast tumors are associated with increased CBC risk. In a population-based case-control study that adjusted for treatment information available in SEER, Saltzman et al. reported that women with ER-/PR- first breast cancer were 60% more likely to be diagnosed with CBC than those with ER+/PR+ tumors, [28] a risk estimate comparable to our findings. In two of the largest and most recent US registry-based studies assessing combined HR status of first tumors and CBC risk, CBC risk was higher among [26] or limited to [25] women with HR- first tumors than among those with HR+ disease, although neither study adjusted for treatment. However, an analysis in the Stockholm Breast Cancer Registry, which included data on endocrine therapy but few other covariates, found no difference in CBC risk by ER status of the first tumor (ER+ standardized incidence ratio (SIR) = 2.30 vs. ER- SIR = 2.17) [29]. PR status was not examined.

Consistent with our findings that women with ER-/PR- first breast cancers are at particularly high risk of ER-/PR- CBC, several other studies have found high concordance between HR status of the first and second primary breast cancers [22, 27, 29, 39]. Whether the high concordance between HR status of the first and second primary breast tumors reflects an underlying genetic susceptibility or an individual’s exposure to hormonal or other risk factors is presently unknown and difficult to determine. It has been established that risk factors for first primary breast cancer vary across breast cancer subtypes defined by HR status or molecular subtypes based on distinct gene expression signatures [40–42]. Thus, phenotypic concordance between two primary tumors may be due, at least in part, to exposure to subtype-specific risk factors [43]. Certain genetic mutations may additionally predispose individuals to developing breast cancer subtypes. In particular, ER- tumors are prevalent among *BRCA1* mutation carriers, [30, 31] which we hypothesize could explain some of the high risk of ER-/PR- CBCs following an ER-/PR- first breast cancer observed in this and in other studies. In our study, the high risk of ER-/PR- CBC following a first tumor with the same phenotype persisted after we excluded *BRCA1/2* mutation carriers. *BRCA1* mutation carriers also tend to develop breast cancer at a relatively young age and other studies have reported that risk of HR- CBC among those with an HR- first tumor was higher among younger women [26, 27, 39]; we did not observe differences by age in our younger population. Furthermore, risks did not appear to differ by breast cancer family history, a possible indicator of underlying genetic susceptibility. It is likely that a combination of both exogenous and endogenous factors, including genetic or molecular factors not measured here, play a role in the development of two cancers with a shared HR-defined phenotype.

Histologic identification of lobular cancer in situ and invasive first breast cancer has frequently been implicated as a risk factor for CBC. Here, we observed a statistically significant 30% increased CBC risk associated with histologic identification of a lobular first tumor, even after adjusting for treatment and other risk factors. This result is consistent with that observed in previous studies [2, 20, 43], although one large population-based study found no elevation in CBC risk comparing lobular with ductal first tumors [21]. Some older studies were conducted prior to the widespread use of endocrine therapy, while a substantial proportion of receptor-positive women in our study received tamoxifen treatment. As virtually all classic lobular tumors are ER+, [44] treatment with tamoxifen may have lowered subsequent CBC risk among women with histologic identification of lobular cancer; accordingly, we observed that histologic identification of lobular cancer was associated with significantly increased CBC risk among women who had not been treated with tamoxifen.

Our study population included women under age 55 years at diagnosis of their first primary tumor. Therefore, the results of this study are most relevant for young women with breast cancer. A limitation of our study is the lack of *BRCA1/2* mutation data for WECARE II Study participants, reducing the statistical power to detect associations among non-carriers. Small numbers of ER+/PR- tumors also limited our ability to examine associations with this subtype. Additionally, HR status was evaluated by the pathology departments of treating hospitals, and was not assessed centrally using a standardized protocol. However, previous work has shown good agreement between HR status reported in cancer registries and HR status determined in a single academic reference laboratory, particularly for ER+/PR+ and ER-/PR- subtypes [45]. Additionally, misclassification of HR status is unlikely to be related to case-control status; as such, any resulting bias will likely be toward the null. We were not able to examine potential effect modification by use of aromatase inhibitors, which lower estrogen levels and have been used in the treatment of ER+ breast tumors in postmenopausal women. Given the relatively young age of participants and because most first breast cancers in our study occurred prior to the widespread use of these drugs, few women in our study population received this therapy. Last, many of the women in our study were diagnosed prior to the identification of HER2 and its incorporation into clinical practice [46]. Therefore, we lacked data on HER2 expression for a large proportion of our participants, affecting our statistical power and limiting our ability to classify tumors into categories that more closely approximate currently recognized molecular subtypes.

## Conclusions

In summary, we observed that HR status and lobular histology of a first breast cancer are predictive of CBC risk. In particular, after adjusting for known CBC risk factors including treatment, women with an ER-/PR- first breast cancer were at a high risk of developing a second breast cancer that was also ER-/PR-. As HR status is a key factor in treatment choice, and ultimately prognosis, these results are informative for risk stratification. Notably, *BRCA1* mutation carriers are known to be at high risk of HR- tumors and CBC, but these associations were also seen in *BRCA1/2* non-carriers. These results suggest that HR status may be useful for informing counseling and screening strategies for CBC risk among women with a first breast cancer. Future work should focus on more refined classification of both first and subsequent primary breast cancers in order to clarify whether these tumors arise from similar etiologic pathways and to identify patients at high risk of CBC.

## Additional file

Additional file 1: Table S1. Association between first breast cancer HR status and CBC, among women not receiving tamoxifen for first diagnosis. In the WECARE Study population of women who had not received tamoxifen for their first breast cancer diagnosis, having an ER-negative first breast cancer or a PR-negative first breast cancer statistically significantly increased the risk of CBC. (DOCX 19 kb)

## Abbreviations

bp: Base pairs
CBC: Contralateral breast cancer
CI: Confidence interval
ER: Estrogen receptor
ER-: Estrogen receptor negative
ER+: Estrogen receptor positive
HER2: Human epidermal growth factor receptor 2
HER2-: Human epidermal growth factor receptor 2 negative
HER2+: Human epidermal growth factor receptor 2 positive
HR: Hormone receptor
PR: Progesterone receptor
PR-: Progesterone receptor negative
PR+: Progesterone receptor positive
RR: Risk ratio
SEER: Surveillance, Epidemiology, and End Results Registry
UBC: Unilateral breast cancer
WECARE: Women’s Environmental Cancer and Radiation Epidemiology Study
