# Supplementary material for: Hormone receptor status of a first primary breast cancer predicts contralateral breast cancer risk in the WECARE study population
Source: Breast Cancer Res. 2017 Jul 19;19:83. doi: 10.1186/s13058-017-0874-x (PMC5517810; doi:10.1186/s13058-017-0874-x)
Supplement: Additional file 1: Table S1. — Association between first breast cancer HR status and CBC, among women not receiving tamoxifen for first diagnosis. In the WECARE Study population of women who had not received tamoxifen for their first breast cancer diagnosis, having an ER-negative first breast cancer or a PR-negative first breast cancer statistically significantly increased the risk of CBC. (DOCX 19 kb) [file 13058_2017_874_MOESM1_ESM.docx]

**Table S1. Association between first breast cancer HR status and CBC, among women not receiving tamoxifen for first diagnosis**

| Receptor Status of First Primary Breast Cancer | Cases, N (%) | Controls, N (%) | RR^a^ (95% CI) |
| --- | --- | --- | --- |
| **ER status** |  |  |  |
| Positive | 407 (39) | 594 (42) | 1.0 (ref.) |
| Negative | 422 (40) | 477 (33) | 1.3 (1.1, 1.6) |
| Other/unknown^b^ | 225 (21) | 354 (25) |  |
|  |  |  |  |
| **PR status** |  |  |  |
| Positive | 336 (32) | 489 (34) | 1.0 (ref.) |
| Negative | 386 (37) | 429 (30) | 1.3 (1.1, 1.7) |
| Other/unknown^b^ | 332 (32) | 507 (36) |  |
|  |  |  |  |
| **Joint ER/PR status** |  |  |  |
| ER+/PR+ | 293 (28) | 404 (28) | 1.0 (ref.) |
| ER+/PR- | 52 (5) | 70 (5) | 1.1 (0.7, 1.7) |
| ER-/PR- | 329 (31) | 341 (24) | 1.4 (1.1, 1.8) |
| Other/unknown^b,c^ | 380 (36) | 610 (43) |  |

Abbreviations: HR: hormone receptor; CBC: contralateral breast cancer; N: number; RR: risk ratio; CI: confidence interval; ER: estrogen receptor; PR: progesterone receptor

^a^ Adjusted for age at first breast cancer diagnosis, first-degree family history of breast cancer, histology, menopausal status, age at menarche, parity, radiation, chemotherapy and hormone therapy at first breast cancer diagnosis.  ^b^Other/unknown’ category consists of women where no lab test was given, the test was given and the results are unknown or the test was given and the results were borderline; estimates not reported. ^c^Includes 39 cases and 73 controls classified as ER-/PR+
